# Programmed environmental illumination during autologous stem cell transplantation hospitalization for the treatment of multiple myeloma reduces severity of depression: A preliminary randomized controlled trial

**DOI:** 10.1002/cam4.1690

**Published:** 2018-08-11

**Authors:** Heiðdís B. Valdimarsdottir, Mariana G. Figueiro, William Holden, Susan Lutgendorf, Lisa M. Wu, Sonia Ancoli‐Israel, Jason Chen, Ariella Hoffman‐Peterson, Julia Granski, Nina Prescott, Alejandro Vega, Natalie Stern, Gary Winkel, William H. Redd

**Affiliations:** ^1^ Psychology Reykjavik University Reykjavik Iceland; ^2^ Population Health Science and Policy Icahn School of Medicine at Mount Sinai New York City New York; ^3^ Rensselaer Polytechnic Institute Troy New York; ^4^ Departments of Psychology and Brain Sciences, Obstetrics and Gynecology, and Urology University of Iowa College of Liberal Arts and Sciences Iowa City Iowa; ^5^ Department of Medical Social Sciences Northwestern University Feinberg School of Medicine Chicago Illinois; ^6^ Department of Psychiatry University of California San Diego La Jolla California

**Keywords:** Autologous Stem Cell Transplant, Bright Light Therapy, Circadian Rhythms, Multiple Myeloma

## Abstract

**Background:**

Over a third of multiple myeloma (MM) patients report clinical levels of depression during autologous stem cell transplant (ASCT) hospitalization. We report preliminary results from a randomized clinical trial investigating the effect of Programmed Environmental Illumination (PEI) of hospital rooms on depression.

**Methods:**

Patients (N = 187) scheduled to receive an ASCT were assessed for eligibility. Those who met study eligibility criteria (n = 44) were randomly assigned to one of two PEI conditions involving delivery of either circadian active bright white light (BWL) or circadian inactive dim white light (DWL) throughout the room from 7 to 10 am daily during hospitalization. Patients completed the Center for Epidemiological Studies Depression Scale (CES‐D) prior to hospitalization, at days 2 and 7 post‐transplant, and on the third day of engraftment.

**Results:**

General linear model analyses revealed no difference between the groups in CES‐D total score at baseline (*P *=* *0.7859). A longitudinal linear mixed model analysis revealed a significant interaction between time of assessment and light condition [*F*(3,107) = 2.90; *P *=* *0.0386; ɳ^2 ^= 0.08)], indicating that PEI prevented the development of depression during hospitalization, with effects reaching significance by the third day of engraftment. At the third day of engraftment, 68.4% of the participants in the DWL comparison condition met the criteria for clinically significant depression compared to 42.1% in the BWL condition.

**Conclusion:**

These findings demonstrate that PEI using BWL during MM ASCT hospitalization is effective in reducing the development of depression. Future studies should examine the mechanisms whereby PEI improves depression.

## 1 INTRODUCTION

Autologous stem cell transplantation (ASCT) is the standard of care for newly diagnosed multiple myeloma (MM) patients determined able to withstand high‐dose chemotherapy.^1^ ASCT hospitalization is associated with high levels of both clinically diagnosed depression and self‐reported depressive symptoms. This depression rises rapidly within the first week of hospitalization.^2^, ^3^, ^4^ One study found that while 7.8% of patients were clinically depressed during the week preceding hospitalization, 36.7% of patients were clinically depressed by day eight of hospitalization.^4^

Depression during ASCT hospitalization has been associated with higher levels of post‐traumatic stress disorder, lower quality of life, and enduring depression in the months and years following the transplant.^5^, ^6^, ^7^ Furthermore, clinical depression following ASCT predicts mortality;^3^, ^8^ one study found that patients with major depression were three times more likely to die between 6 and 12 months following ASCT than those without major depression.^8^

Despite the high levels of depression during ASCT hospitalization and its adverse effects on psychological adjustment and mortality following ASCT, depression is underdiagnosed and undertreated among MM patients.^9^ Moreover, current approaches to treating depression during ASCT hospitalization are limited. Pharmacological interventions, such as antidepressant medications, pose potential risks for interaction with specific drugs used during the ASCT and show little benefit over placebo.^10^ Although nonpharmacological interventions such as cognitive behavioral therapy and exercise are effective in treating depression in cancer patients,^11^, ^12^ they are extremely difficult to implement during ASCT hospitalization due to severe patient debility. Patients are rendered emotionally and physically exhausted; they have little interest in food, television, or even conversing with family members.

An easy to deliver, low‐burden and inexpensive intervention to alleviate depression is light therapy. Light therapy has been extensively used to treat seasonal affective disorder (SAD),^13^ and two recent meta‐analyses showed that light therapy is effective in reducing nonseasonal depression as well.^14^, ^15^ One hypothesized mechanism through which light therapy reduces depression involves the promotion of circadian entrainment. Individuals suffering from depression exhibit circadian disruption such as disturbances in the timing of sleep, melatonin, and cortisol rhythms.^16^ Cancer and its treatment are also associated with circadian disruption.^17^, ^18^, ^19^ Given that light is one of the strongest *zeitgebers* for the circadian system, it is logical to hypothesize that a lighting system designed to promote circadian entrainment in a hospital setting may reduce depressive symptoms.

Previous studies have administered bright white light (BWL) in various forms including handheld light boxes that deliver 2500 lux at the eye for 2 hours or 10 000 lux at the eye for 30 minutes to treat both seasonal and nonseasonal depression.^13^, ^15^ The retinal mechanisms underlying circadian phototransduction (ie, how the retina converts light signals into electrical signals for the master clock and regulates circadian rhythms) have recently been elucidated. It is now well established that suppression of melatonin synthesis, a marker of the clock, is maximally sensitive to short‐wavelength light with a peak around 460 nm.

Rea and colleagues proposed a mathematical model of human circadian phototransduction based upon published spectral sensitivity data,^20^, ^21^ retinal neurophysiology, and neuroanatomy, including the operating characteristics of circadian phototransduction, ranging from response threshold to saturation.^22^, ^23^, ^24^ According to the Rea model, spectral irradiance at the cornea is first converted into circadian light (CL_A_) reflecting the spectral sensitivity of the circadian system that is then transformed into a circadian stimulus (CS). The CS value reflects the absolute sensitivity of the circadian system. Thus, CS is a measure of the *effectiveness* of retinal light to stimulate the human circadian system, as measured by acute melatonin suppression, from threshold (CS = 0.1 or 10% melatonin suppression) to saturation (CS = 0.7, or 70% melatonin suppression). It is important to note that CL_A_ and CS characterize the spectral and absolute sensitivities of light‐induced nocturnal melatonin suppression via the biological clock. CL_A_ and CS are assumed to characterize the spectral and absolute sensitivities of the entire human circadian system because the suprachiasmatic nucleus (SCN) is the central link in a wide variety of daily regulatory functions such as hormone production and sleep. For the purpose of this study, it was accepted that the spectral and absolute sensitivities of nocturnal melatonin suppression are similar to those controlling light‐induced changes of circadian timing, or circadian entrainment.

Disrupted circadian rhythms co‐occur with depression, both of which manifest through insomnia and early morning awakening. Indeed, these are common diagnostic criteria for clinical depression.^16^ Sleep propensity rhythms closely associate with endogenous melatonin rhythms, and melatonin acts as both a soporific (sleep‐promoting), and chronobiotic (circadian‐entraining) agent. It is unclear, however, whether depressed individuals exhibit increased or decreased melatonin secretion, with studies supporting both notions.^25^ Interestingly, the antidepressant action of exogenous melatonin has been robustly demonstrated in animal studies.^25^, ^26^, ^27^ Agomelatine, a widely used antidepressant and structural derivative of melatonin, is a melatonin receptor (MT1 & MT2) agonist as well as a serotonin receptor (5‐HT_2c_) antagonist. Whether its antidepressant action can be attributed to a synergistic effect or to one of its receptor interactions independently is unknown.^28^ Melatonin's relationship to depressed mood may instead be due to its chronobiotic activity. A healthy endogenous melatonin rhythm may be necessary, but not sufficient, to relieve depression with underlying circadian rhythm disruption. Melatonin, whether endogenous or exogenous, will synchronize the SCN; therefore, it is imperative to control its timing in order to entrain circadian rhythms. This can be accomplished using light therapy.

In the present study, we investigated the use of a novel ambient lighting intervention, Programmed Environmental Illumination (PEI) using freestanding light fixtures that delivered either Bright White Light (BWL) or Dim White Light (DWL) daily during hospitalization. The BWL delivered a CS ≥ 0.30 at the eye between 7 and 10 am daily during hospitalization. A similar lighting system delivering a CS ≥ 0.30 showed a significant improvement in sleep, mood, and behavior in our field research with Alzheimer's disease patients and other populations.^23^, ^29^, ^30^, ^31^ Relative to other light delivery systems, such as light boxes or light goggles, PEI requires no patient effort; the patient is able to remain passive in bed as the circadian stimulating light illuminates the room.

The present research investigates the impact of delivering circadian active light (CS = 0.3 BWL) from 7 to 10 am to MM patients during their ASCT hospital stay compared to patients receiving circadian inactive light (CS = 0.1, DWL). We hypothesized that participants exposed to the BWL would experience lower levels of depression during hospitalization than those exposed to the DWL. These analyses are part of a larger program of research examining the effects of PEI in treating cancer‐related fatigue during and following ASCT.

## 2 METHODS

### 2.1 Participants

**Figure 1:**
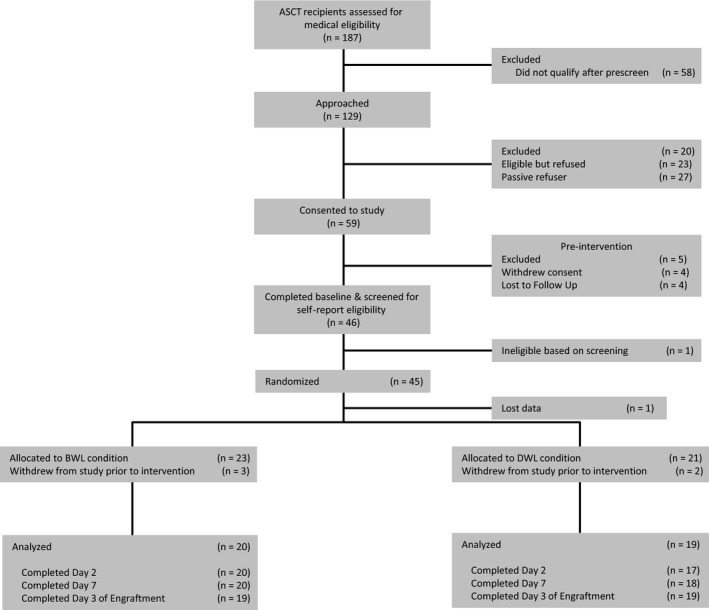
CONSORT: trial participant flow.

Participant flow through the study is shown in Figure 1. Data were analyzed from 44 MM patients undergoing ASCT at Mount Sinai Hospital in New York, NY. Eligibility criteria included: first ASCT; 21 years of age or older; and English language proficiency. Exclusion criteria included: previous stem cell transplantation (autologous or allogeneic), pregnancy, eye diseases limiting the patient's ability to process light (eg, untreated cataracts, severe glaucoma, macular degeneration, blindness, pupil dilation problems, or retina damage), secondary cancer diagnosis within the last 5 years, severe sleep disorders (eg, Narcolepsy), history of bipolar disorder or manic episodes (a contra‐indication for light treatment), severe psychological impairment (eg, hospitalization for depressive episode in the past 12 months), and/or previous use of light therapy to alleviate fatigue or depressive symptoms. The Mt. Sinai Program for the Protection of Human Subjects approved the study.

### 2.2 Procedure

Study personnel approached MM patients during their regular clinical visits. Study personnel described the study, obtained signed consent and administered the screening/eligibility measures questionnaires. Participants who qualified for the study were randomized using a block randomization table to either the bright white light (BWL; n* *=* *23) or the dim white light comparison group (DWL; n* *=* *21) group.^32^ Following randomization, an un‐blinded study coordinator programmed each light fixture to the correct light condition prior to installation. All conditions were concealed from both the patient and the blinded study coordinators who administered the psychological questionnaires and communicated with patients. Patients completed the Center for Epidemiological Studies Depression scale (CES‐D) independently using a study iPad. Exceptions were made for patients experiencing debilitating fatigue, in which case the surveys were administered orally. Blinded coordinators did not enter the hospital rooms while the light fixtures were turned on. Study participants completed CES‐D at four timepoints: baseline, prior to hospitalization, day 2 post‐transplant, day 7 post‐transplant, and the third day (approximately day 14 post‐transplant) of engraftment. Engraftment was defined as the period where the patient's absolute neutrophil count is above 500 cells/mm^3^. After three consecutive days above this value, the patient is considered fully engrafted; this is a marker of successful transplantation required for discharge.

### 2.3 Programmed environmental illumination (PEI)

**Figure 2:**
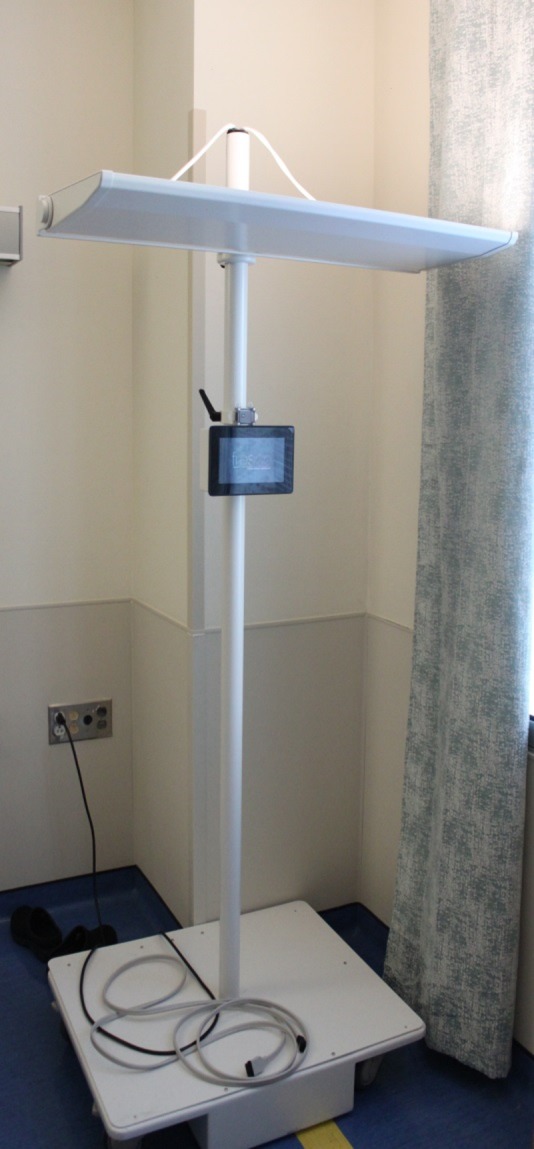
Freestanding light fixture

**Figure 3:**
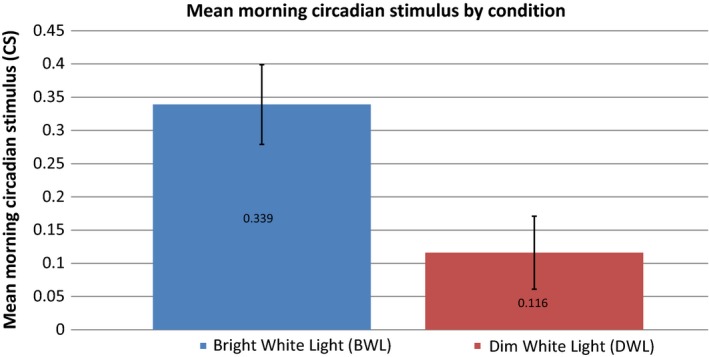
Mean ± Standard Deviation of CS obtained from the bed Daysimeters values included are from the hours when the lighting intervention was programmed to be energized. As hypothesized, there was a significant difference in CS values received in the morning between the two groups

Acuity Brands provided freestanding light fixtures (Figure 2) delivering a CS value of 0.3 for the active, bright white light (BWL) condition (1300 lux at the eye level of the patients) and a CS of 0.1 for the dim white light (DWL) condition (90 lux at the eye level of the patients). Therefore, the BWL was designed to deliver circadian active light while the DWL was designed to deliver circadian inactive light. Ambient light illuminated the entire room. As is shown in Figure 3, both BWL (*M* = 0.339, SEM = 0.06) and DWL (*M* = 0.116, SEM = 0.055) were successful in delivering light at the intended CS values. The light fixtures, previously programmed to the respective light condition, remained in the patients' hospital rooms for the duration of hospitalization and were calibrated to turn on every morning from 7 to 10 am. During this time, patients were instructed to go about their normal activities, which could include leaving their hospital room. Based on prior research, 30 minutes a day of circadian active bright white light was sufficient in reducing fatigue.^33^ A 3‐hour time frame was chosen to ensure at least 30 minutes of exposure regardless of activity from 7 to 10 am. In order to verify that the correct dose of light was received, Daysimeters, light meters calibrated to measure CS, were placed behind the patient's bed, on the light fixture, and on their chest, worn as a pendant by the patient during waking hours for the entire hospital stay. The Daysimeter is a research tool manufactured by the Lighting Research Center at Rensselaer Polytechnic Institute; they are not commercially available.^34^ As hypothesized, those in the BWL condition received light of a significantly (*P* < 0.0001) higher CS value than those in the DWL (Figure 3).

### 2.4 Measures

Depressive symptoms were measured using the 20‐item Center for Epidemiologic Studies Depression Scale (CES‐D).^35^ A cutoff score of 16 or greater identifies individuals at risk for clinical depression. The CES‐D has been shown to be a reliable measure for assessing the number, types, and duration of depressive symptoms across racial, gender, and age categories (Cronbach's alpha ≥0.85). Concurrent validity by clinical and self‐report criteria as well as substantial evidence of construct validity has been demonstrated. The CES‐D was reliable in the present study (Cronbach's alpha = 0.81, N = 44) and has been established as a valid and reliable measure of depression among cancer patients.^36^

### 2.5 Statistical analysis

Using an intent‐to‐treat analysis and data from all randomized participants, the BWL condition was compared to a DWL condition. To determine equivalence between the BWL and DWL groups at baseline, a general linear model (GLM) was conducted. For the main analyses, a repeated‐measures linear mixed model (LMM) was used to evaluate change in depressive symptomatology over time using a random intercepts model. This approach makes use of all the data that each patient provided in parameter estimation and significance testing, even if some of the repeated measures are missing.

Although the present research represented a trial effort to determine the effect size that would guide power considerations in a larger study, we used the results of Redd et al^33^ to obtain an estimate of the effect size for the time by treatment condition interaction associated with the depression measure in the present pilot investigation. In the Redd et al^33^ study of fatigue in stem cell transplantation survivors, the interaction effect size was 0.98, indicating that a sample size of 60 patients would be sufficient to obtain a good estimate of effect size for power using the data from the present study.^37^ In the present report, 44 patients were evaluated as part of an interim analysis.

## 3 RESULTS

### 3.1 Participant characteristics

Table 1 summarizes the sample's descriptive demographic and medical data, indicating that most study participants were male (59%), relatively well educated (65% reported a college or graduate degree), upper income (50% reported incomes above $80 000 per year), and either married or in a marriage equivalent (64%). The majority (59%) were Caucasian, and 27% self‐identified as Black or African American. At the time of transplant, the majority of participants (54%) had reached a very good partial response to treatment, were not on depression medications (91%), and were stage I (50%) on the International Staging System (ISS). Using the HCT comorbidity index, 59% had scores at 2 points or below. Importantly, there were no differences between the groups on demographic or medical variables (*P* < 0.2).

**Table 1:** Patient demographic and medical variables

	BWL	DWL
	n / (%)	n / (%)
Gender (n = 44)	n = 23	n = 21
Male	14 (61)	12 (57)
Female	9 (39)	9 (43)
Race/Ethnicity (n = 44)	n = 23	n = 21
Black or African‐American	9 (39)	3 (14)
White	12 (52)	14 (67)
Other	2 (9)	4 (19)
Marital status (n = 44)	n = 23	n = 21
Not Married	10 (43)	6 (29)
Currently married/marriage equivalent	13 (57)	15 (71)
Children at home (n = 40)	n = 21	n = 19
No	15 (71)	8 (42)
Yes	6 (29)	11 (58)
Education (n = 43)	n = 23	n = 20
High school or less	4 (17)	5 (25)
Partial college	2 (9)	4 (20)
College graduate	17 (74)	11 (55)
Employed (n = 44)	n = 23	n = 21
No	13 (57)	12 (57)
Yes	10 (43)	9 (43)
Annual income (in dollars) (n = 40)	n = 20	n = 20
Below 80 000	8 (40)	12 (60)
Above 80 000	12 (60)	8 (40)
Pre‐HCT disease status (n = 44)	n = 23	n = 21
No evidence of disease	0 (0)	0 (0)
Complete response	1 (4)	1 (5)
Near complete response	1 (4)	1 (5)
Very good partial response	14 (61)	10 (48)
Partial response	6 (26)	6 (28)
Stable disease	1 (4)	1 (5)
Progressive disease	0 (0)	2 (9)
HCT comorbidity index total score (n = 44)	n = 23	n = 21
0	4 (17)	4 (19)
1	2 (9)	1 (5)
2	8 (35)	7 (33)
3	4 (17)	4 (19)
4	0 (0)	1 (5)
5	1 (4)	2 (9)
6	1 (4)	1 (5)
7	2 (9)	1 (5)
8	1 (4)	0 (0)
Depression medication Pre‐HCT	n = 23	n = 21
No	21 (91)	19 (90)
Yes	2 (9)	2 (10)
ISS stage	n = 23	n = 21
1	13 (57)	9 (42)
2	1 (4)	5 (24)
3	1 (4)	1 (5)
Unspecified	8 (35)	6 (29)

Table reports tracked demographic and medical variables and their prevalence within the sample. All demographic information was gathered through self‐report questionnaires. All medical information was gathered through medical chart screening, including a pretransplant physician assessment that included the HCT comorbidity score, ISS stage and status of disease. Chi‐square analyses revealed no significant differences between the groups on any demographic or medical variables.

### 3.2 Expectancy

The credibility/expectancy scale was administered at baseline for all participants and at day 2 post‐transplant and third day of engraftment (both following patient exposure to conditions) for a subset of the participants. All data were analyzed for differences. There were no significant differences between the BWL and DWL groups at baseline [*F*(1,41) = 0.723; *P *=* *0.400], day 2 post‐transplant [*F*(1,11) = 0.010; *P *=* *0.923], and on the third day of engraftment [*F*(1,11) = 0.494; *P *=* *0.497].

### 3.3 Depressive symptoms

**Figure 4:**
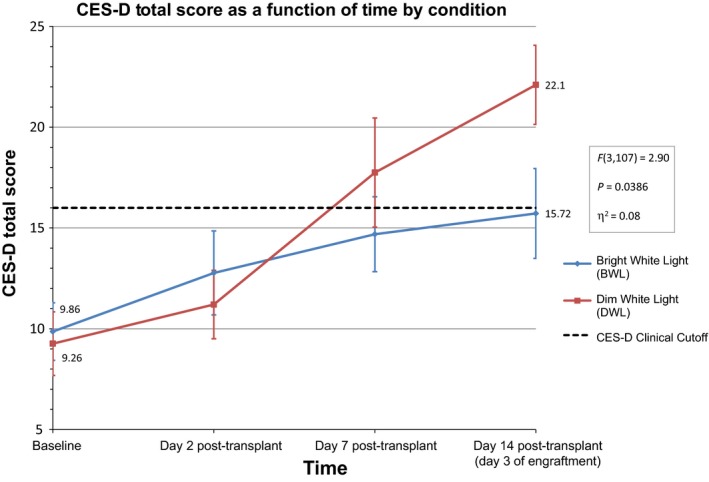
Mean ± Standard Deviation of CES‐D scores obtained at each time point. As hypothesized, there was a significant difference in CES‐D total scores by Day 14 post‐transplant (day 3 of engraftment).

The GLM analysis of the CES‐D total score revealed no significant difference in depressive symptoms between the BWL (n = 23, *M* = 9.26, SD = 7.03) and DWL (n = 21, *M* = 9.86, SD = 7.43) groups at baseline (*P* = 0.7859). The repeated‐measures LMM indicated that the main effect of time on CES‐D total score was significant [*F*(3,110) = 13.35; *P* < 0.0001] . Specifically, there was an increase in depressive symptoms for all study participants during transplant hospitalization (Mean Baseline = 9.54; Mean Day 2—Post‐Transplant = 12.09; Mean Day 7—Post‐Transplant = 16.18; Mean Day 14—Engraftment = 18.97). The interaction of the time by treatment condition effect was significant [*F*(3,107) = 2.90; *P* = 0.0386], with a medium effect size (ɳ^2 ^= 0.08). This result means that changes in depression over time differed depending on whether the patient was exposed to either the DWL or BWL condition. A graphical plot of the interaction (Figure 4) illustrates how the BWL and DWL groups differed in their trajectories of depressive symptoms over the study period. Figure 4 shows a greater increase in depressive symptoms for patients in the DWL condition compared to patients in the BWL condition beginning at day 7 post‐transplant and peaking on the third day of engraftment. Additionally, Figure 4 shows that the mean levels of depression were below the clinical depression cutoff of 16 on the CES‐D scale in the BWL group at all timepoints, while this was not the case in the DWL group.

### 3.4 Clinical depression

At baseline, 28.6% and 17.4% of the participants in the DWL and BWL, respectively, were above the clinical depression cutoff of 16 on the CES‐D. At day 7 post‐transplant, about 40% of participants in both groups scored above the clinical cutoff. However, at the third day of engraftment, 68.4% of participants in the DWL group scored above the clinical cutoff compared to 42.1% of the participants in the BWL group.

## 4 DISCUSSION

The present study examined the effectiveness of Programmed Environmental Illumination (PEI) using freestanding light fixtures in the hospital room delivering circadian active bright white light (BWL) from 7 to 10 am daily to reduce depressive symptoms during ASCT. PEI using BWL reduced the severity of depressive symptoms during hospitalization compared to circadian inactive dim white light (DWL). To our knowledge, this is the first study to show beneficial effects of PEI on depressive symptoms among MM patients undergoing ASCT.

The BWL and DWL groups differed in both their trajectory and final levels of depressive symptoms during hospitalization. Mean levels of depressive symptoms increased sharply during the first week of hospitalization in both groups, then leveled off in the BWL group but continued to increase in the DWL condition until the third day of engraftment. Mean levels of depression were below the clinical depression cutoff on the CES‐D scale in the BWL group at all timepoints. However, the DWL group remained above the clinical cutoff beginning at day 7.

Depressive symptoms that met the CES‐D clinical cutoff criteria for depression showed a similar pattern. In both the BWL and the DWL groups, the proportion of patients meeting the clinical cutoff criteria increased from approximately 20% at baseline to approximately 40% at day 7 post‐transplant. The proportion of patients meeting the depression cutoff criteria then continued to increase in the DWL group, but remained stable the BWL group with 68.4% and 42.1% meeting the depression cutoff criteria, respectively, at the third day of engraftment.

It is important to draw attention to the impact of PEI on depression in relation to how sick ASCT patients are during their hospitalization. As mentioned in the introduction, their exhaustion renders them able to do very little as their hospitalization continues. Both patients and physicians attribute the hospitalization period with the highest degrees of distress, often viewing it as unmodifiable.^38^, ^39^ The capability of PEI to prevent the worsening of depressive symptoms indicates real clinical benefit.

The discovery of a clinically significant benefit from PEI in the prevention of escalating depression adds to the growing literature demonstrating that BWL can effectively treat depressive symptoms in diverse populations.^22^, ^40^, ^41^, ^42^ These results have the potential for major clinical implications as depressive symptoms during hospitalization affect not only patient quality of life^5^, ^7^ but also increases their risk of subsequent morbidity and mortality.^8^

The findings may also have major implications for lighting in hospital rooms. Circadian active ambient light in the hospital room may not only reduce depressive symptoms but also other common symptoms of circadian disruption experienced by hospitalized cancer patients including fatigue and sleep disturbances. Our previous studies have shown that systematic light exposure delivering BWL by light book reduced fatigue and improved sleep efficiency among cancer survivors.^33^, ^43^ We also found that PEI reduced depression in Alzheimer's disease patients.^22^, ^23^, ^24^ Future studies should include comprehensive measures of common side effects during hospitalization as well as biological measures of circadian rhythm disruption to allow for examination of the mechanisms whereby light might impact these side effects.

The results reported here must be interpreted in the context of its limitations. First, the study is preliminary with a relatively small sample size and is based on a larger program of research examining the effects of light to treat cancer‐related fatigue. Second, the results presented here did not include post‐hospitalization assessments of depressive symptoms. It is not yet known if PEI using BWL during hospitalization affects depressive symptoms during the post‐transplant period. Third, the potential mechanisms underlying the effects of BWL on depressive symptoms were not examined; however, our primary hypothesis is that circadian entrainment plays a role.

The present findings demonstrate that an easy to deliver, low cost intervention alleviates depression during hospitalization for autologous stem cell transplantation. The light intervention may have application across a wide range of clinical settings and patient populations.

## CONFLICT OF INTEREST

No conflicts of interest to disclose.
